# Autoimmune thyroid disease as a risk factor for angioedema in patients with chronic idiopathic urticaria: a case-control study

**DOI:** 10.1590/S1516-31802012000500005

**Published:** 2012-11-13

**Authors:** Ruy Felippe Brito Gonçalves Missaka, Henrique Costa Penatti, Maria Regina Cavariani Silvares, Célia Regina Nogueira, Gláucia Maria Ferreira da Silva Mazeto

**Affiliations:** I Undergraduate Medical Student, Department of Internal Medicine, Botucatu Medical School, Universidade Estadual Paulista (Unesp), Botucatu, São Paulo, Brazil.; II MD, PhD. Professor of Dermatology, Department of Dermatology and Radiotherapy, Botucatu Medical School, Universidade Estadual Paulista (Unesp), Botucatu, São Paulo, Brazil.; III MD, PhD. Professor of Endocrinology and Metabology, Department of Internal Medicine, Botucatu Medical School, Universidade Estadual Paulista (Unesp), Botucatu, São Paulo, Brazil.; IV MD, PhD. Professor of Endocrinology and Metabology, Internal Medicine Department, Botucatu Medical School, Universidade Estadual Paulista (Unesp), Botucatu, São Paulo, Brazil.

**Keywords:** Angioedema, Allergy and immunology, Autoimmunity, Hashimoto disease, Thyroiditis, Urticaria, Angioedema, Alergia e imunologia, Auto-imunidade, Doença de Hashimoto, Tireoidite, Urticária

## Abstract

**CONTEXT AND OBJECTIVE:**

An association between chronic idiopathic urticaria (CIU) and autoimmune thyroid disease (ATD) has been reported. However, there have not been any reports on whether ATD raises the risk of angioedema, which is a more severe clinical presentation of CIU. Thus, the aim of the present study was to evaluate whether the risk of angioedema is increased in patients with CIU and ATD.

**DESIGN AND SETTING:**

Case-control study including 115 patients with CIU at a tertiary public institution.

**METHODS:**

The patients were evaluated with regard to occurrence of angioedema and presence of ATD, hypothyroidism or hyperthyroidism.

**RESULTS:**

Angioedema was detected in 70 patients (60.9%). There were 22 cases (19.1%) of ATD, 19 (16.5%) of hypothyroidism and nine (7.8%) of hyperthyroidism. The risk among patients with ATD was 16.2 times greater than among those without this thyroid abnormality (confidence interval, CI = 2.07-126.86). The odds ratio for hypothyroidism was 4.6 (CI = 1.00-21.54) and, for hyperthyroidism, 3.3 (CI = 0.38-28.36).

**CONCLUSIONS:**

Patients with CIU and ATD presented greater risk of angioedema, which reinforces the idea that a relationship exists between this allergic condition and thyroid autoimmunity. This finding could imply that such patients require specifically directed therapy.

## RESUMO

**CONTEXTO E OBJETIVO:**

A associação de urticária crônica idiopática (UCI) com doença autoimune da tireoide (DAT) é relatada. Porém, não foram encontrados relatos se a DAT eleva o risco de angioedema, uma apresentação clínica mais grave da UCI. Assim, o objetivo do presente estudo foi avaliar se o risco de angioedema está aumentado em pacientes com UCI e DAT.

**TIPO DE ESTUDO E LOCAL:**

Estudo caso-controle, incluindo 115 pacientes com UCI em uma instituição pública terciária.

**MÉTODOS:**

Os pacientes foram avaliados quanto à ocorrência de angioedema e à presença de DAT, hiper ou hipotireoidismo.

**RESULTADOS:**

Angioedema ocorreu em 70 pacientes (60,9%). Foram observados 22 (19,1%) casos de DAT, 19 (16,5%) de hipotireoidismo e 9 (7,8%) de hipertireoidismo. Os pacientes com DAT apresentaram risco 16,2 vezes maior de angioedema do que os sem a alteração tireoidiana (intervalo de confiança, IC = 2.07-126.86). O *odds ratio*, para hipotireoidismo, foi de 4,6 (IC = 1.00-21.54) e para hipertireoidismo foi de 3,3 (IC = 0.38-28.36).

**CONCLUSÕES:**

Pacientes com UCI e DAT apresentaram maior risco de angioedema, reforçando a ideia de existência de relação entre o quadro alérgico e autoimunidade tireoidiana. Este achado poderia implicar em um direcionamento terapêutico específico para tais pacientes.

## INTRODUCTION

Chronic urticaria (CU) is a skin disorder characterized by the appearance of itchy, erythematous swellings that persist for longer than six weeks.^1^ It most commonly affects middle-aged women, and is associated with angioedema in up to 40% of the cases. Angioedema is considered to be the most severe clinical manifestation of this disorder and may be associated with a risk of death.^2^^,^^3^ The causes of CU are multiple and complex. It is named chronic idiopathic urticaria (CIU) in approximately 70% of the cases, since no cause can be identified. However, clinical-laboratory^4^^,^^5^ and histopathological studies^6^^,^^7^ have suggested that CU has autoimmune etiology at least in a subset of patients.

The thyroid gland is a frequent target of autoimmune diseases such as Graves’ disease (GD) and Hashimoto’s thyroiditis (HT), which can cause hyperthyroidism and hypothyroidism, respectively.^8^

Since autoimmune diseases may have common etiopathogenic mechanisms, an association between CIU and autoimmune thyroid diseases (ATD) is theoretically possible. Indeed, higher levels of anti-thyroid antibodies and thyroid dysfunctions have been reported in CIU patients since 1983.^9^ Moreover, some authors have suggested that these patients have a more prolonged and severe course, and respond poorly to treatment,^10^^,^^11^^,^^12^^,^^13^ with occurrence of cases of angioedema in the presence of thyroid autoimmunity.^14^ However, no data have yet been produced regarding whether ATD increases the risk of angioedema. Such data could contribute towards clarifying the complex relationship between thyroid autoimmunity and CIU.

## OBJECTIVES

The aim of this study was to assess whether the risk of angioedema is higher in patients with CIU and ATD.

## METHODS

### Study design and size

We conducted a case-control study that included all 115 CIU patients attended at a tertiary public institution between 1984 and 2006. Demographic characteristics (age, gender and self-reported skin color) and the presence of angioedema, ATD, hyperthyroidism and hypothyroidism were assessed. The patients with and without angioedema were compared with regard to demographic data. In addition, patients with thyroid abnormalities were evaluated for the risk of angioedema. The study was approved by the Ethics Committees of Botucatu Medical School, São Paulo State University (Universidade Estadual Paulista, Unesp) (number 2406/2007).

### Measurements and laboratory tests

CIU was defined as a situation of having four or more episodes of hives per week that had not been triggered by any external allergen, over a period of at least six weeks. At our center, etiological investigation of CIU is conducted rigorously, as recommended in the literature.^15^ Patients with other types of urticaria, contact eczema, or systemic autoimmune diseases, such as systemic lupus erythematosus and scleroderma, were excluded. The diagnosis of angioedema was based on compatible history, i.e. the presence of inflammatory edema in the face, tongue, larynx or extremities.^2^

Thyroid abnormalities were diagnosed based on levels of thyrotropin (TSH), free thyroxine (FT4), anti-thyroperoxidase (anti-TPO) and anti-thyroglobulin (anti-TG) antibodies, measured by means of chemiluminescence (DPC, Los Angeles, United States). Thyroid function was classified as normal when TSH and FT4 were within normal reference ranges (0.4-4.0 mIU/ml and 0.8-1.9 ng/dl, respectively); hypothyroid, when TSH was elevated; and hyperthyroid, when TSH was suppressed. Hypothyroidism and hyperthyroidism were considered to be overt when FT4 levels were not within the normal range; or subclinical when FT4 was normal. Independently of other abnormalities found, the presence of anti-TPO and/or anti-TG on two different occasions was deemed indicative of ATD. Thus, for analysis purposes, thyroid abnormalities were classified as ATD, hypothyroidism or hyperthyroidism.

### Statistical analysis

The mean ages of the cases with and without angioedema were compared using Student’s t test, and the frequencies of other parameters studied were compared using the chi-square test. Statistical significance was set at 5.0%. The odds ratio was estimated using the Woolf method, with a 95% confidence interval.^16^

## RESULTS

The patients presented a mean age of 36.5 years (range from 30.0 to 49.0 years). Most of them were females (79.1%) and considered themselves to be white (87.2%).

There were 22 cases (19.1%) of ADT, 19 (16.5%) of hypothyroidism (16 subclinical) and nine (7.8%) of hyperthyroidism (five subclinical). Among the hypothyroidism cases, 17 presented HT; while among the hyperthyroidism cases, five were diagnosed as presenting GD.

There were 70 cases of angioedema (60.9%). Differences in age, gender and skin color between patients with and without angioedema were not statistically significant (Table 1).

**Table 1.** Demographic characteristics of 115 patients with chronic idiopathic urticaria (CIU), with and without angioedema

Demographic characteristic	Angioedema	
	Yes n = 70 (60.9%)	No n = 45 (39.1%)
Age (years)^*^	37.97 ? 12.44	36.83 ? 14.19
Male (%)^†^	15.7	27.8
Caucasian (%)^†^	85.3	90.9

n = number of patients. ^*^Mean ? standard deviation; P = 0.642 (Student’s t test for independent samples). ^†^Chi-square test; P > 0.05.

**Figure 1.**
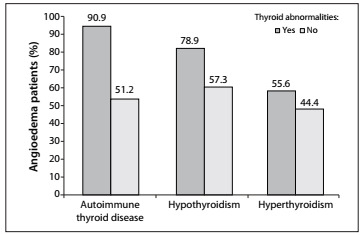
Percentage distribution of 115 chronic idiopathic urticaria (CIU) patients, with and without thyroid abnormalities, according to whether they presented angioedema.

The percent rate of angioedema was higher in patients with thyroid abnormalities than in patients without thyroid abnormalities (Figure 1).

Among the patients with ATD, the chance of having angioedema was 16.2 times higher (confidence interval, CI = 2.07-126.86). Among the patients with hypothyroidism and hyperthyroidism, the chances of having angioedema were, respectively, 4.6 and 3.3 times higher, but these differences were not statistically significant (P > 0.05; Table 2).

**Table 2.** Chance of angioedema in patients with chronic idiopathic urticaria (CIU) and thyroid abnormalities (95% confidence interval)

Thyroid abnormality	Odds ratio	Confidence interval (95%)
Autoimmune disease	16.2	(2.07-126.86)^*^
Hypothyroidism	4.6	(1.00-21.54)
Hyperthyroidism	3.3	(0.38-28.36)

^*^Statistically significant (P < 0.05).

## DISCUSSION

According to some authors, CIU and thyroid disorders are more common among women than among men.^1^^,^^17^ Consistent with these reports, more women than men participated in the present study (3.79 females for each male). The mean age of our patients was 36.5 years, which is similar to what was reported by other authors,^15^^,^^17^^,^^18^ and most patients had white skin. This is in agreement with Silvares et al. who, in an assessment on CIU patients from the same area, found that most of them were Caucasian (94%) and female (3:1). In addition, they observed that CU affected patients at all ages, but particularly those between 20 and 50 years (mean age of about 35 years).^3^

Hypothyroidism was the most frequent dysfunction in our sample (16.5%). Pimenta et al. found a similar rate (16%) in outpatients.^19^ However, the older age of their patients might have caused the prevalence of hypothyroidism to be higher. In another study conducted in Rio de Janeiro, the hypothyroidism rate was 12.3% among 1220 individuals.^20^

The frequency of hyperthyroidism was 7.8%. CIU may be associated with thyroid hormone excess. In fact, high thyroid hormone levels have been reported to lead to immunological disorders, probably due to changes in suppressor T lymphocytes.^21^ Some previous studies have reported dermatological improvement in patients with hyperthyroidism after treatment with anti-thyroid drugs, but this has not been corroborated by others. Moreover, most studies have just been case reports or included a small sample.^22^^,^^23^^,^^24^ Thus, the relationship between hyperthyroidism and CIU remains to be established.

ATD was detected in 19.1% of the patients with CIU, in agreement with other authors.^10^^,^^15^^,^^18^^,^^25^ The association between CIU and ATD has long been recognized as significant,^9^^,^^10^^,^^11^^,^^17^^,^^25^^,^^26^ notwithstanding biasing factors such as ATD diagnostic bias,^26^ lack of a control group^10^ and small sample size.^25^ The pathophysiology of the association is not well understood, but it seems that anti-thyroid antibodies are not directly responsible for the lesions seen in patients and only serve as an indicator of autoimmunity.^27^ Interest in this topic has increased, because it has been reported that urticaria improves with normalization of thyroid function.^22^^,^^23^^,^^24^^,^^27^

In agreement with other reports,^9^^,^^17^^,^^25^ HT was the most frequently detected ATD (77.3%) in our patients, and was observed in 14.8% of the CIU cases. Camargo et al. found a similar rate of chronic thyroiditis among 829 individuals from a general population in the city of São Paulo (17.6%).^28^

The frequency of angioedema in the CIU group (60.9%) was higher than what was reported by others. Feibelmann et al. observed angioedema in 49.97% of their patients with CIU,^15^ while others have reported rates of about 40%.^13^ Silvares et al. evaluated CU patients in the same area as where the present study was performed, and detected angioedema in 51.8% of the cases.^3^ The reason why a higher frequency of angioedema was found in our patients remains unclear, but it may be associated with regional iodine sufficiency, or with the fact that the ATD rate found in this study was higher (19.1%) than what was reported by Feibelmann et al. (12.3%),^15^ and higher than in American studies on the general population (3-10%).^8^^,^^29^

The association between angioedema and ATD was evident. Even though there is a scarcity of reports in the literature confirming this association, CIU associated with ATD is believed to have a more prolonged and severe course.^10^^,^^12^^,^^13^ Nonetheless, based on the frequency of the crises and associations with mucosal angioedema and histamine resistance, Verneuil et al. did not find higher CU severity levels in the presence of ATD,^26^ which was in agreement with Feibelmann et al.^15^ Other studies have reported high prevalence of ATD in patients with hereditary angioedema^30^ and improvement of isolated angioedema in patients treated with thyroxin, thus suggesting that there may be an association between ATD and angioedema.^31^ Nonetheless, neither of these studies included any CIU cases, and therefore they cannot be properly compared with ours. In a prospective investigation on 139 patients, Toubi et al. suggested that long-lasting CU was associated with the presence of anti-thyroid antibodies (ATA) and angioedema. In their study, 45% of the patients with angioedema and 12% of the patients without angioedema still had CU, and 52% of the ATA-positive patients and 16% of the ATA-negative patients were still suffering from CU after five years of follow-up. However, these authors did not assess the association between CU and ATD, thus making it impossible to compare their findings with those reported here.^11^

The findings from the present study provide a stimulus for investigating the possible cause-and-effect relationship between thyroid autoimmunity and CIU and the potential pathophysiological mechanisms involved in this process. Furthermore, these findings serve as a warning that greater care needs to be taken with patients presenting an association of CIU with ATD, regarding the risk of angioedema, which is a more severe form of urticaria that requires emergency treatment, particularly when it affects the glottis.

## CONCLUSION

ATD increased the risk of developing angioedema, which is a more severe form of CIU, thus reinforcing the idea that CIU and ATD (particularly HT) are related. This finding could imply that such patients require specifically directed therapy.
